# Structural Features and Mitogenome-Based Evolutionary Insights into *Acanthopleura loochooana* (Polyplacophora: Chitonidae)

**DOI:** 10.3390/ijms27073053

**Published:** 2026-03-27

**Authors:** Xinyue Que, Qiong Wu, Yifan Zou, Peng Xiang, Guangcheng Chen, Longjiang Mao, Bingpeng Xing

**Affiliations:** 1 School of Marine Sciences, Nanjing University of Information Science & Technology, Nanjing 210044, China; 2 Third Institute of Oceanography, Ministry of Natural Resources, Xiamen 361005, China; 3 Naval Architecture and Ocean Engineering, Kunsan National University, Gunsan 54150, Republic of Korea

**Keywords:** mitogenome, Polyplacophora, molecular phylogeny, Chitonidae, comparative mitogenomics

## Abstract

*Acanthopleura loochooana* is a widely distributed intertidal chiton in the northwestern Pacific, yet its mitochondrial genomic architecture and evolutionary position within Chitonidae have not been comprehensively evaluated. In this study, we sequenced and analyzed the complete mitochondrial genome of *A. loochooana* using next-generation sequencing. The mitogenome is a circular double-stranded DNA molecule of 15,295 bp that contains the typical 37 mitochondrial genes, including 13 protein-coding genes (PCGs), 22 transfer RNA genes, and 2 ribosomal RNA genes. Codon usage patterns show a clear preference for A- or U-ending codons, consistent with trends observed in other polyplacophoran mitogenomes. Phylogenetic analyses based on concatenated sequences of the 13 mitochondrial PCGs under Bayesian frameworks recovered congruent topologies with strong nodal support. *A. loochooana* was placed in a well-supported clade with *Liolophura japonica* and *A. vaillantii*, providing a reference within Acanthopleurinae. These results provide the complete mitochondrial genome of *A. loochooana* and contribute new mitogenomic data to the currently limited dataset of Polyplacophora, offering additional insights into mitochondrial genome organization and phylogenetic relationships within Acanthopleurinae.

## 1. Introduction

Chitons have a long evolutionary history, with early chiton-like lineages traceable to the Late Cambrian–Early Ordovician, whereas the crown-group Polyplacophora was likely established in the Carboniferous [1,2]. Members of this class possess a characteristic eight-plated shell and a relatively conservative body plan, which have made them important taxa for investigating early molluscan evolution and character transformation [3]. Within Polyplacophora, the family Chitonidae is among the most diverse and widely distributed, occurring primarily in intertidal and shallow rocky habitats. Species in this family typically graze on algal biofilms using their radula and are regarded as key components of intertidal benthic communities [4]. Several deep-sea chitons, such as *Leptochiton boucheti* (Sirenko, 2001), *Leptochiton foresti* (Leloup, 1981), and *Nierstraszella lineata* (Nierstrasz, 1905), have been reported from sunken-wood habitats in the West Pacific. These species typically occur at depths of 200–1500 m and inhabit decomposing wood substrates on the seafloor. They are considered microbial grazers that feed on bacterial and fungal biofilms that develop on wood surfaces and often form dense local aggregations in wood-fall ecosystems [5].

*Acanthopleura loochooana* (Broderip & G. B. Sowerby I, 1829) belongs to the class Polyplacophora, order Chitonida, and family Chitonidae, and is a representative species of the genus *Acanthopleura.* It is commonly found on intertidal rocky shores in Hainan (China), Japan, and surrounding coastal regions. This species has frequently been recorded in regional biodiversity and ecological surveys, yet detailed genomic information for this taxon remains scarce [6].

Although chitons have long attracted attention in evolutionary and systematic studies, molecular genomic resources remain limited for many species within Chitonidae, particularly for complete mitochondrial genome data. Mitochondrial genomes are widely used in animal phylogenetics because of their conserved gene content, usually maternal inheritance, and relatively rapid evolutionary rate [7]. In Polyplacophora, mitochondrial genomes are generally characterized by a conserved gene order and the apparent absence of a typical control region [8], yet comparative evaluations across closely related taxa remain insufficient. Furthermore, phylogenetic relationships within Acanthopleurinae, including the taxonomic placement of genera such as *Acanthopleura* and *Liolophura*, have been discussed based on morphological and limited molecular evidence, but mitogenomic support remains sparse [6,7,9,10]. Nevertheless, most available data are restricted to a small number of taxa. For *A. loochooana*, previous research has largely focused on morphology and ecology, while its mitochondrial genome organization and phylogenetic placement have not been thoroughly investigated. The lack of such genomic information has constrained further exploration of evolutionary relationships within Chitonidae and patterns of mitochondrial genome evolution in Polyplacophora.

In this study, we sequenced and analyzed the complete mitochondrial genome of *A. loochooana* and performed a comprehensive evaluation of its genomic organization, nucleotide composition, and codon usage patterns. We further conducted phylogenetic analyses using concatenated mitochondrial protein-coding genes and compared the newly obtained sequence with available mitogenomic data from related polyplacophoran taxa. By integrating structural and evolutionary perspectives, this study provides a systematic assessment of the mitogenome of *A. loochooana* and contributes to a broader understanding of mitochondrial genome evolution and phylogenetic relationships within Chitonidae.

## 2. Results

### 2.1. Structure and Composition of the Mitogenome

**Figure 1:**
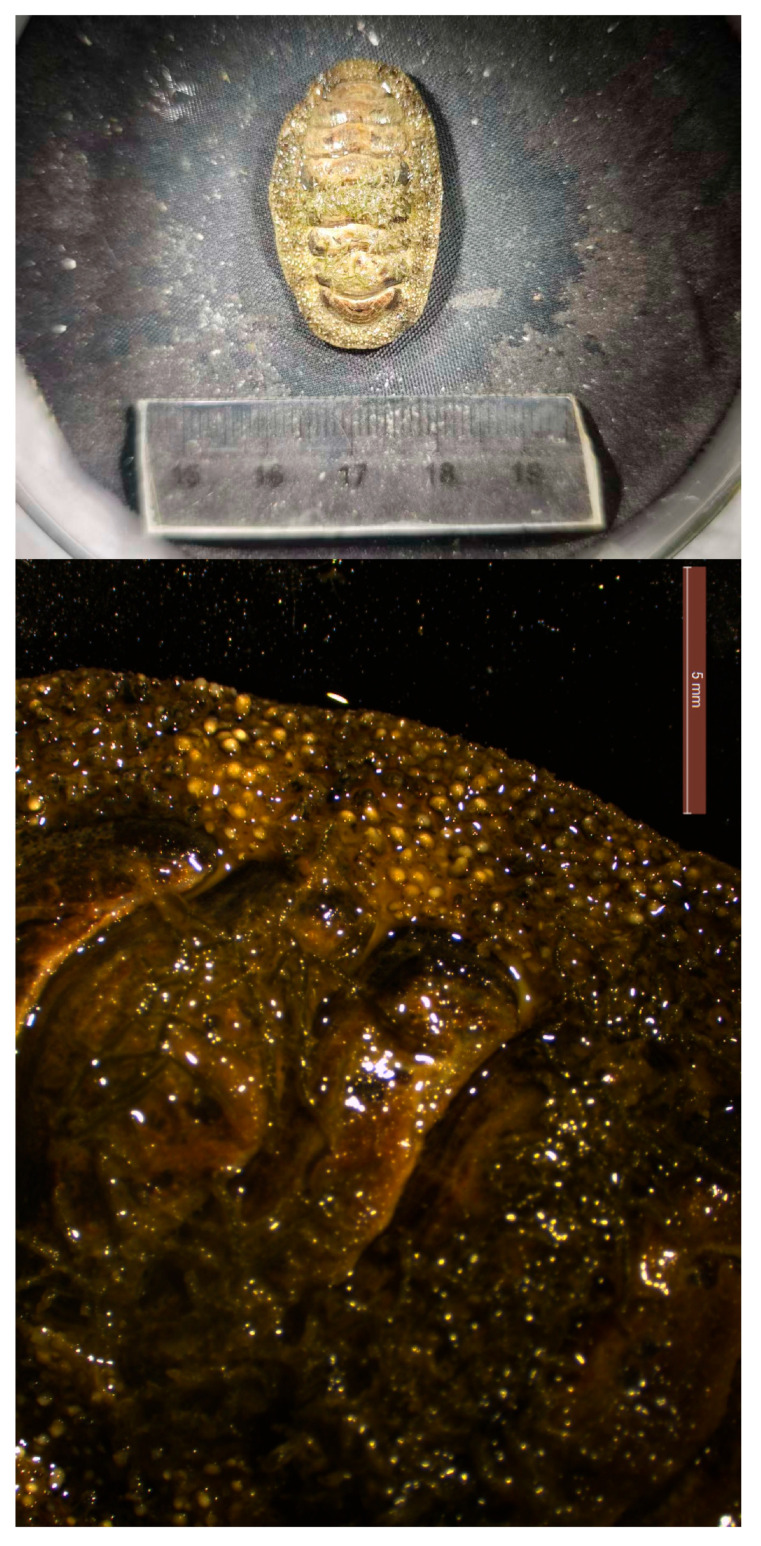
*Acanthopleura loochooana* and its girdle in detail.

**Figure 2:**
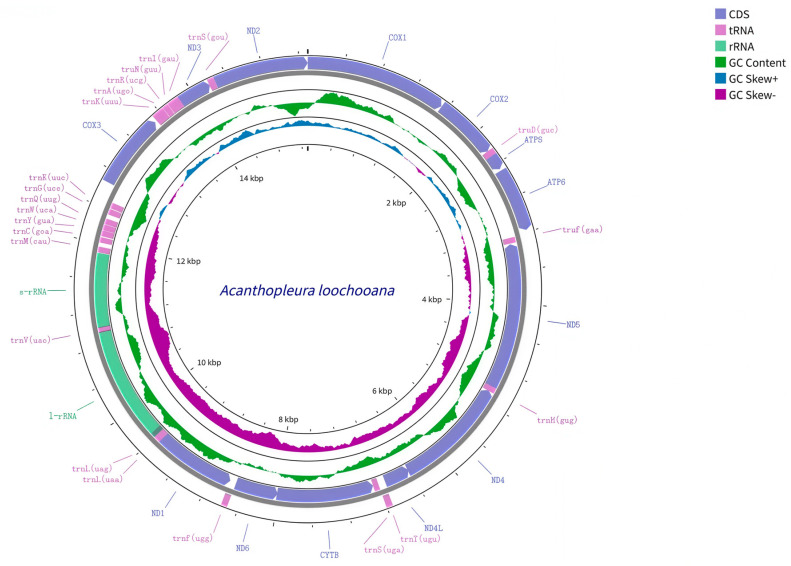
Mitogenome map of *Acanthopleura loochooana.* Note: The orientation of the arrows for each gene (CDS, tRNA, rRNA) represents the direction of transcription. Clockwise arrows signify genes encoded on the positive strand, while counterclockwise arrows indicate those on the negative strand. The colored rings represent coding genes (CDS, purple), transfer RNA (tRNA, pink), and ribosomal RNA (rRNA, green), respectively. The innermost ring indicates GC content (blue) and GC skew (GC Skew+/-, purple).

The species identity of *A. loochooana* (Figure 1) was confirmed based on diagnostic morphological characters described in previous studies [6,7], and was further supported by COI sequence similarity to reference sequences available in GenBank. The mitogenome of *A. loochooana* is circular, with a total length of 15,295 bp (Figure 2). It comprises 13 protein-coding genes, 2 rRNA genes, and 22 tRNA genes. Among these 37 genes, 13 are located on the heavy strand (H): COX1, COX2, ATP8, ATP6, COX3, trnK(UUU), trnA(UGC), trnR(UCG), trnN(GUU), trnI(GAU), ND3, trnS(GCU), and ND2. The remaining 24 genes are encoded on the light strand (L). Additionally, we identified 20 intergenic spacers (length range: 1–181 bp) and 10 gene overlaps (length range: 1–58 bp). The longest intergenic spacer is located between COX3 and trnE(UUC), whereas the longest gene overlap occurs between 16S rRNA (rrnL) and trnL(UAG) (Table 1). The gene order of this mitogenome is consistent with that of other species in the genus *Acanthopleura* [11].

### 2.2. Protein-Coding Genes and Codon Usage

The complete length of the protein-coding genes (PCGs) in *A. loochooana* was determined to be 11,221 bp, accounting for 73.4% of the total mitogenome sequence. As shown in Table 1, only six PCGs (ND1, ND4, ND4L, ND5, ND6, and CYTB) were located on the light strand (L-strand), while the remaining PCGs were all located on the heavy strand (H-strand). This distribution pattern on the L- and H-strands is consistent with that observed in *Schizochiton incisus* (G. B. Sowerby II, 1841). Ten of the 13 PCGs use ATG as the start codon and TAA as the stop codon. In contrast, ND1 and ND4 used GTG as the start codon, with TAA and TAG as the stop codons, respectively. ND2 initiates with TTG and terminates with TAA. Among the 13 PCGs, the A + T content was 69.7%, whereas the G + C content was 30.2%, indicating a strong A + T bias in the protein-coding genes of *A. loochooana*. Nucleotide compositional asymmetry was assessed using AT-skew = (A − T)/(A + T) and GC-skew = (G − C)/(G + C). The AT-skew and GC-skew values of the PCGs were −0.202 and 0.145, respectively, suggesting a higher proportion of T over A and G over C.

**Figure 3:**
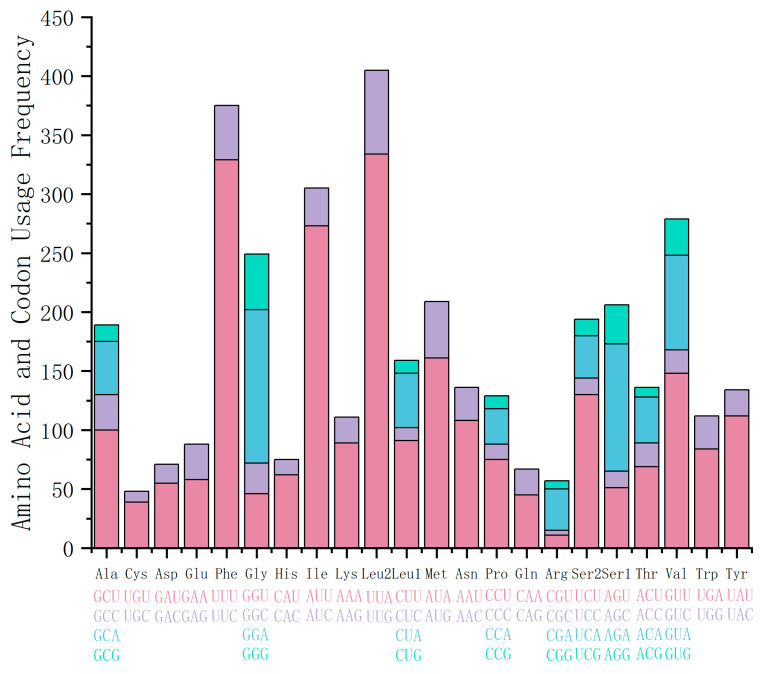
Amino Acid and Codon Usage Frequencies in the Protein-Coding Genes of the *Acanthopleura loochooana* mitogenome. Note: Amino acids are provided on the *x*-axis, with their corresponding synonymous codons listed below each abbreviation. The total usage count of each amino acid is shown on the *y*-axis. The stacked segments within each bar represent the relative usage of different codons encoding the same amino acid.

During the translation of genes into proteins, organisms often exhibit a bias toward using specific codons more frequently than others, a phenomenon known as codon usage bias [12,13]. In the 13 PCGs of *A. loochooana*, a total of 3728 codons were identified (excluding start codons). The most frequently used amino acids were Leu2, Phe, and Ile, while Cys was the least frequent. Among codons, UUA, UUU, and AUU were the most frequently utilized (Figure 3). The results indicate that codons ending in A or U were the most common, and RSCU values for NNU and NNA codons were generally greater than 1.

### 2.3. Phylogenetic Analysis of the Mitogenome

**Figure 4:**
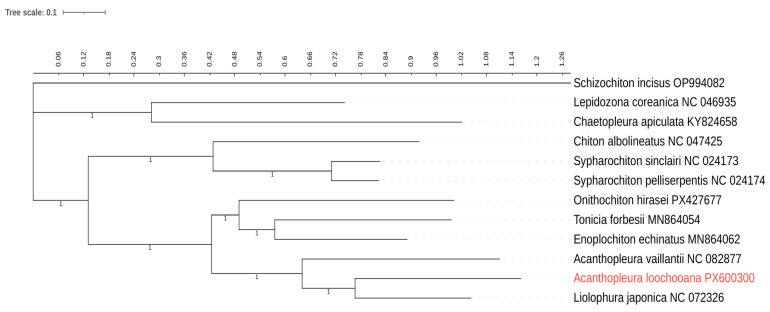
Phylogenetic tree of Chitonida inferred from 13 mitochondrial protein-coding genes (PCGs) using Bayesian inference (BI) analyses. Node values represent Bayesian posterior probabilities.

As shown in Figure 4, the phylogenetic tree exhibits a clearly resolved branch structure, and all nodes are supported with posterior probabilities of 1.00. The phylogenetic tree is primarily divided into three major clades, corresponding to two families and one subfamily. The results indicate that *L. japonica* (Lischke, 1873) and *A. loochooana* are most closely related, followed by the relationship between *A. vaillantii* (Rochebrune, 1882) and *A. loochooana*. The subfamily containing *Onithochiton hirasei* (Pilsbry, 1901), *Tonicia forbesii* (Carpenter, 1857), and *Enoplochiton echinatus* (Barnes, 1824) is closely related to both *A. loochooana* and the Acanthopleurinae subfamily to which it belongs.

## 3. Discussion

### 3.1. Mitogenome Organization and Structural Characteristics of A. loochooana

The mitochondrial genome of *A. loochooana* exhibits a circular structure and a complete set of genes typical of Polyplacophora. Its genome size (15,295 bp) and gene order are highly similar to those reported for other chitonid species, indicating strong conservation of mitochondrial architecture at the family level [14]. Such structural stability has been repeatedly observed in chitons and contrasts with the extensive gene rearrangements documented in several other molluscan groups, suggesting that distinct evolutionary constraints may act on polyplacophoran mitogenomes [8].

In addition, the presence of multiple intergenic spacers and overlapping genes reflects the compact organization typical of animal mitochondrial genomes [10]. The longest intergenic spacer was located between trnE and COX3. This position coincides with the region where a putative control region has been identified in other polyplacophoran species. Therefore, the corresponding non-coding region in *A. loochooana* may represent the homologous putative control region, although its precise functional significance remains unclear. Broader comparative analyses across Chitonidae will be necessary to determine whether variation in non-coding regions shows lineage-specific patterns or carries phylogenetic signal.

### 3.2. Protein-Coding Genes and Codon Usage Bias

Protein-coding genes account for more than 70% of the mitochondrial genome of *A. loochooana* and exhibit a pronounced A + T bias, consistent with mitochondrial genomes of other mollusks. The observed negative AT-skew and positive GC-skew indicate strand-specific nucleotide asymmetry, which is commonly attributed to asymmetric replication processes in mitochondrial DNA [15].

Codon usage analysis revealed a clear preference for codons ending in A or U, with UUA, UUU, and AUU among the most frequently used. This pattern closely mirrors the underlying nucleotide composition of the mitogenome, indicating that mutational bias plays a primary role in shaping synonymous codon usage [16]. At the same time, deviations from uniform codon usage suggest that selective constraints related to translational efficiency and tRNA availability may also contribute to codon preference. Comparable codon usage patterns have been reported in other chiton mitogenomes, pointing to a conserved trend across Polyplacophora rather than lineage-specific adaptation [17]. Collectively, these results suggest that codon usage bias in *A. loochooana* is driven by the combined effects of long-term mutational pressure and purifying selection, consistent with the functional constraints expected for mitochondrial protein-coding genes.

### 3.3. Phylogenetic Implications for Chitonidae and Acanthopleurinae

Mitochondrial genomes have become an important source of data for reconstructing phylogenetic relationships within Polyplacophora. In the present study, phylogenetic analysis based on concatenated mitochondrial protein-coding genes recovered a well-resolved topology with high Bayesian posterior probabilities across major nodes. *A. loochooana* was recovered as closely related to *L. japonica* and *A. vaillantii*, in agreement with previous morphological and molecular evidence [10].

The recovered relationships provide independent mitogenomic support for recent taxonomic proposals advocating the inclusion of *Liolophura* within *Acanthopleura* [18]. Although this hypothesis has previously been supported mainly by anatomical evidence and nuclear gene analyses, the present results indicate that mitochondrial genomes also reflect this evolutionary relationship, further supporting the recognition of Acanthopleurinae as a cohesive lineage within Chitonidae [19,20,21].

Nevertheless, the uniformly high support values observed in mitochondrial phylogenies should be interpreted with caution. Early diversification within Chitonidae may have involved rapid lineage splitting, potentially accompanied by incomplete lineage sorting [22]. Moreover, mitochondrial genomes represent a single, maternally inherited locus and may not fully capture evolutionary processes such as introgression or historical gene flow. Consequently, while mitochondrial data provide robust resolution for shallow and intermediate relationships, resolving deeper divergences within Chitonidae will require integrative approaches incorporating nuclear genomic data and detailed morphological assessments.

### 3.4. Limitations and Future Perspectives

Several limitations of this study should be considered. The mitogenome of *A. loochooana* was obtained from a single specimen collected at one locality, preventing an assessment of intraspecific variation and population-level mitochondrial diversity. In addition, phylogenetic inference relied exclusively on mitochondrial protein-coding genes, which represent only a subset of the genomic information relevant to evolutionary history. Because mitochondrial genomes are maternally inherited and represent a single genetic locus, they may not fully reflect deeper evolutionary processes such as incomplete lineage sorting, introgression, or historical gene flow. Finally, taxon sampling remains constrained by the limited availability of complete mitogenomes for Polyplacophora, particularly within Chitonidae.

Future research should prioritize expanded geographic sampling and population-level analyses of *A. loochooana* and related taxa, as well as the integration of nuclear genomic markers. Such integrative datasets will be essential for detecting cryptic diversity, refining species boundaries, and achieving a more comprehensive understanding of evolutionary patterns and processes in chitons. In addition, the present phylogenetic analysis was based on nucleotide sequences rather than amino acid sequences. Although nucleotide data are informative for relatively shallow divergences, amino acid-based analyses may be useful for evaluating deeper relationships and should be explored in future studies.

## 4. Materials and Methods

### 4.1. Sample Collection and DNA Extraction

The specimen of *A. loochooana* was collected in Pingtan, Fuzhou, China (Voucher specimen was deposited at the Third Institute of Oceanography, Ministry of Natural Resources, under the number PX600300. Contact person: Bingpeng Xing, bluprin@tio.org.cn. Coordinates: 119.88° E, 25.57° N). The specimens were preliminarily identified based on the morphological characteristics of *A. loochooana* [23], preserved in 75% ethanol, and stored at −20 °C for subsequent analyses. Subsequently, approximately 20–30 mg of muscle tissue was taken, and genomic DNA was extracted using the commercial Vazyme FastPure kit (Vazyme, Nanjing, China). PCR amplification of a partial mitochondrial cytochrome c oxidase subunit I gene (COI) fragment was performed using the primer pair dgHCO2198 (forward) and dgLCO1490 (reverse) [24]. The annealing temperature was set at 45 °C, increasing by 0.5 °C per cycle for 15 cycles, followed by 49 °C for 20 cycles. Reactions were carried out in a 25 μL volume, and gradient PCR was applied to determine the optimal annealing temperature. The resulting COI sequence was used solely for preliminary species identification and was not included in mitogenome assembly or subsequent phylogenetic analyses.

### 4.2. Sequencing and Annotation of the Mitogenome

Next-generation sequencing was performed by Novogene Co., Ltd. (Novogene, Tianjin, China). A paired-end library was constructed using the Illumina TruSeq™ DNA Sample Preparation Kit and sequenced on the Illumina NovaSeq 6000 platform (Illumina, Inc., San Diego, CA, USA), generating 150 bp paired-end reads. The sequencing data showed high quality, with a Q30 ratio above 90%, and provided sufficient depth for mitochondrial genome assembly. Raw read quality was assessed using FastQC v0.11.5, and adapter sequences and low-quality reads were removed using fastp v0.23 [25]. The filtered reads were assembled into the complete mitochondrial genome using GetOrganelle v1.7.5.0 with a k-mer size of 31. Default parameters were used for all other settings. Circularization of the mitogenome was first assessed during the assembly process and then verified by examining terminal overlap consistency, completeness of the mitochondrial gene set, and the correctness of start and stop codons in protein-coding genes. Genome annotation was performed using the MITOS2 online tool (https://usegalaxy.org/) [26] under the invertebrate mitochondrial genetic code. The positions of protein-coding genes, transfer RNAs, and ribosomal RNAs were identified accordingly. Relative synonymous codon usage (RSCU) values and codon usage frequencies were calculated using PhyloSuite v1.2, and the resulting data were visualized using Origin 2024.

### 4.3. Phylogenetic Tree Construction

The phylogenetic tree was constructed from the newly sequenced mitogenome of *A. loochooana* and nine other chitonid mitogenomes downloaded from NCBI GenBank, with *S. incisus* selected as the outgroup (Table 2). Sequence preprocessing and model selection prior to tree construction were performed using PhyloSuite v.1.2. First, the 13 protein-coding sequences were extracted [27,28], followed by sequence alignment using MAFFT v.7.313 [29]. The aligned sequences were subsequently trimmed using Gblocks v0.91 (default parameters) to eliminate poorly aligned regions. The concatenated dataset of the 13 mitochondrial protein-coding genes was analyzed as a single partition. ModelFinder v2.2.0 [30] was used to select the best-fit substitution model, and GTR+F+I+G4 was identified as the optimal model. Bayesian inference was then performed using MrBayes v3.2 [31], for 2,000,000 generations, with the first 25% of sampled trees discarded as burn-in.

## 5. Conclusions

This study presents the complete mitochondrial genome of *A. loochooana*, thereby expanding the still-limited mitogenomic resources available for Polyplacophora. The mitogenome exhibits a conserved gene complement and organization typical of chitons, with a compact structure, strong A+T bias, and codon usage patterns broadly consistent with those reported in other members of Chitonidae. These features provide useful comparative data for understanding mitochondrial genome evolution in chitons. Phylogenetic analysis based on concatenated mitochondrial protein-coding genes placed *A. loochooana* together with *L. japonica* and *A. vaillantii* within Acanthopleurinae, providing additional mitogenomic evidence relevant to relationships within this subfamily. However, because mitochondrial genomes are maternally inherited and represent a single locus, caution is needed when interpreting deeper phylogenetic relationships. Overall, the newly generated mitogenome of *A. loochooana* provides a valuable reference for future integrative studies combining mitochondrial, nuclear, and morphological data to further clarify chiton phylogeny and evolutionary history.

## Figures and Tables

**Table 1:** Mitogenome Composition of *Acanthopleura loochooana*.

Locus	Position	Size (bp)	Intergenic Region (bp)	Start Coding	Stop Coding	Strand
trnM(cau)	1–68	68	3			L
trnC(gca)	116–179	64	47			L
trnY(gua)	192–261	69	12			L
trnW(uca)	263–327	65	1			L
trnQ(uug)	332–400	69	4			L
trnG(ucc)	452–517	66	51			L
trnE(uuc)	527–595	69	9			L
COX3	777–1598	822	181	ATG	TAA	H
trnK(uuu)	1626–1694	69	27			H
trnA(ugc)	1691–1759	69	−4			H
trnR(ucg)	1763–1824	62	3			H
trnN(guu)	1829–1892	64	4			H
trnI(gau)	1893–1957	65	0			H
ND3	1958–2314	357	0	ATG	TAA	H
trnS(gcu)	2313–2380	68	−2			H
ND2	2384–3403	1020	3	TTG	TAA	H
COX1	3404–4936	1533	0	ATG	TAA	H
COX2	4945–5637	693	8	ATG	TAA	H
trnD(guc)	5636–5703	68	−2			H
ATP8	5704–5865	162	0	ATG	TAA	H
ATP6	5888–6583	696	22	ATG	TAA	H
trnF(gaa)	6625–6691	67	41			L
ND5	6698–8413	1716	6			L
trnH(gug)	8414–8478	65	0			L
ND4	8474–9826	1353	−5	GTG	TAA	L
ND4L	9820–10,122	303	−7	ATG	TAG	L
trnT(ugu)	10,137–10,207	71	14			L
trnS(uga)	10,207–10,271	65	−1			L
CYTB	10,275–11,414	1140	3	ATG	TAA	L
ND6	11,407–11,913	507	−8	ATG	TAA	L
trnP(ugg)	11,916–11,984	69	2			L
ND1	11,987–12,928	942	2	GTG	TAA	L
trnL(uaa)	12,929–12,995	67	0			L
trnL(uag)	12,996–13,062	67	0			L
l-rRNA	13,005–14,387	1381	−58			L
trnV(uac)	14,370–14,436	67	−18			L
s-rRNA	14,429–15,295	867	−8			L

**Table 2:** List of Species and Genomic Information for Phylogenetic Analysis in the Order Chitonida.

Order	Family	Genus	Species	Accession No.	Reference
Chitonida	Chitonidae	*Acanthopleura*	*A. loochooana*	PX600300	This study
			*A. vaillantii*	NC082877	Unpublished
		*Liolophura*	*L. japonica*	NC072326	Unpublished
		*Enoplochiton*	*Enoplochiton echinatus*	MN864062	Irisarri et al., 2020 [8]
		*Tonicia*	*T. forbesii*	MN864054	Irisarri et al., 2020 [8]
		*Onithochiton*	*O. hirasei*	PX427677	Unpublished
		*Sypharochiton*	*Sypharochiton pelliserpentis*	NC024174	Veale et al., 2016 [32]
		*Sypharochiton*	*Sypharochiton sinclairi*	NC024173	Veale et al., 2016 [32]
		*Chiton*	*Chiton albolineatus*	NC047425	Unpublished
	Chaetopleuridae	*Chaetopleura*	*Chaetopleura apiculata*	KY824658	Unpublished
	Ischnochitonidae	*Lepidozona*	*Lepidozona coreanica*	NC046935	Unpublished
	Schizochitonidae	*Schizochiton*	*S. incisus*	OP994082	Unpublished

## Data Availability

The original contributions presented in this study are included in the article. Further inquiries can be directed to the corresponding author.
